# Controversies in the management of asymptomatic carotid stenosis: from best medical therapy to a redefinition of surgical indications

**DOI:** 10.3389/fneur.2026.1875031

**Published:** 2026-07-02

**Authors:** Jianghao Zhou, Wei Zhu, Yong Lu, Huiming Dou, Guochu Peng, Changyang Zhong

**Affiliations:** 1 Zhejiang Chinese Medical University, Hangzhou, China; 2 Hangzhou Hi-tech Industry Development Zone (Binjiang District) Bureau of Health, Hangzhou, China; 3 Hangzhou Binjiang District Puyan Street Community Health Service Center, Hangzhou, China; 4 Hangzhou Binjiang District Xixing Street Community Health Service Center, Hangzhou, China; 5 Hangzhou Binjiang District Changhe Street Community Health Service Center, Hangzhou, China; 6 Cerebrovascular Disease Department, Hangzhou Third People's Hospital, Hangzhou, China

**Keywords:** asymptomatic carotid stenosis, best medical therapy, carotid artery stenting, stroke, transcarotid artery revascularization

## Abstract

Asymptomatic carotid artery stenosis has become increasingly detected with population aging and the widespread use of high-resolution vascular imaging. Its management is undergoing a transition from stenosis-based anatomical assessment toward multidimensional biological risk stratification. The relative benefit of prophylactic carotid revascularization, including carotid endarterectomy and carotid artery stenting, versus best medical therapy remains controversial. In recent years, best medical therapy has evolved substantially with intensive lipid-lowering strategies, PCSK9 inhibitors, novel glucose-lowering agents, and improved control of vascular risk factors, thereby reshaping the expected benefit of preventive intervention. Meanwhile, recent randomized trials, including ACST-2, ECST-2, and CREST-2, have challenged the traditional reliance on luminal stenosis as the dominant criterion for intervention. This review summarizes key evidence from 2021 to 2026 regarding contemporary medical therapy, revascularization trials, and emerging approaches to risk assessment in asymptomatic carotid stenosis. It discusses the clinical value of high-resolution magnetic resonance imaging, cerebrovascular hemodynamic reserve, and exosomal microRNAs for identifying vulnerable plaques and biologically high-risk disease. It also reconsiders the definition of “asymptomatic” by incorporating covert cognitive impairment and findings from CREST-H. Finally, this review proposes an individualized precision-management framework based on multimodal biomarkers.

## Introduction

### Epidemiological shifts and the current dilemma in clinical decision-making

Asymptomatic carotid artery stenosis (aCAS) refers to atherosclerotic narrowing of the proximal carotid artery confirmed by Doppler ultrasonography or other imaging methods. It is diagnosed in patients who have not had any transient ischemic attack (TIA), amaurosis fugax, or ischemic stroke related to the ipsilateral carotid circulation within the previous 6 months. The management of aCAS has changed markedly over the past few decades. From a pathophysiological perspective, aCAS is a local manifestation of systemic atherosclerosis at the carotid bifurcation under disturbed blood flow. The main mechanisms include endothelial dysfunction, lipid deposition, macrophage infiltration, and smooth muscle cell proliferation, which eventually lead to progressive luminal narrowing. Although it is classified as “asymptomatic,” this condition is neither benign nor static. Epidemiological studies show that the prevalence of aCAS continues to increase with population aging. Among individuals older than 60 years, the prevalence of aCAS with stenosis ≥50% is estimated to be 4.2 to 7.5%, while in men older than 80 years, it may exceed 30%. This condition is often accompanied by severe coronary artery disease and a high rate of all-cause mortality. Given the large number of affected individuals, identifying an optimal intervention strategy with both clinical benefit and health-economic value has become a major public health challenge in cerebrovascular medicine (1, 2).

Two landmark randomized controlled trials (RCTs) conducted in the 1990s, the Asymptomatic Carotid Atherosclerosis Study (ACAS) and the Asymptomatic Carotid Surgery Trial (ACST-1), established carotid endarterectomy (CEA) as a major treatment option for severe aCAS (3). In ACAS, the estimated 5-year risk of ipsilateral stroke decreased from 11.0% in the medical therapy group to 5.1% in the surgical group, corresponding to an absolute annual benefit of approximately 1%. However, this benefit was observed only in women, not in men, and it took 2 years for the surgical advantage to emerge after accounting for periprocedural risks. The number needed to treat (NNT) to prevent one stroke over 5 years was 17. In ACST-1, the perioperative risk of stroke and death was 3%, and no benefit of surgery was observed in patients older than 75 years. These historical limitations are essential for contextualizing the contemporary debate on prophylactic revascularization. Despite these limitations, ACAS and ACST-1 established a durable benchmark for perioperative risk (<3%) that remains embedded in current guidelines (e.g., 2022 SVS, 2023 ESVS), and their long-term follow-up continues to inform the natural history of severe aCAS. Our review therefore uses the 2021–2026 window to capture the most recent evolution of best medical therapy and revascularization trials, while referencing these foundational studies where relevant. These early high-level studies showed that, in patients with stenosis ≥60%, prophylactic CEA could significantly reduce the long-term risk of ipsilateral disabling or fatal ischemic stroke. In ACAS, for example, the estimated 5-year risk of stroke decreased from 11.0% in the medical therapy group to 5.1% in the surgical group, corresponding to a relative risk reduction of 53%. Based on these findings, prophylactic revascularization was widely incorporated into international clinical guidelines and became the standard interventional approach for severe aCAS. Despite these historical limitations, ACAS and ACST-1 established a durable benchmark for perioperative risk (<3%) that remains embedded in current guidelines (e.g., 2022 SVS, 2023 ESVS), and their long-term follow-up continues to inform the natural history of severe aCAS. Our review therefore uses the 2021–2026 window to capture the most recent evolution of best medical therapy and revascularization trials, while referencing these foundational studies where relevant.

However, when these classical trials are reassessed from the perspective of modern evidence-based medicine, their historical limitations become more apparent. Both ACAS and ACST-1 were conducted in the pre-statin era. At that time, medical treatment was largely limited to low-dose aspirin and basic antihypertensive therapy. High-intensity statin treatment and strict metabolic control were not yet part of routine care. As a result, the relatively high annual stroke rate in the control groups of these early trials, approximately 2 to 3%, likely reflected the natural course of atherosclerosis without effective intervention rather than the true effect of contemporary standardized medical therapy.

In recent years, major advances in cardiovascular pharmacotherapy have greatly expanded the concept of best medical therapy (BMT). Contemporary BMT is now a comprehensive vascular protection strategy. It includes potent antiplatelet therapy, novel targeted lipid-lowering treatments such as PCSK9 inhibitors, intensive blood pressure and glycemic control with agents such as SGLT-2 inhibitors and GLP-1 receptor agonists, and sustained lifestyle intervention. Several recent large-scale cohort studies have shown that, in patients with aCAS receiving standardized high-intensity BMT, the annual incidence of ipsilateral ischemic stroke has fallen to 1.0%, and in some reports to below 0.5%. This marked reduction in risk has raised serious questions about the conventional indications for prophylactic revascularization. If modern BMT can reduce stroke risk to such a low level, the periprocedural risk associated with surgery or endovascular intervention may offset, or even exceed, its long-term preventive benefit. In this context of ongoing clinical uncertainty, the present systematic review aims to critically appraise the most recent evidence published between 2021 and 2026, address the main controversies in the management of aCAS, and explore a precision medicine pathway based on multimodal biomarkers.

**Figure 1:**
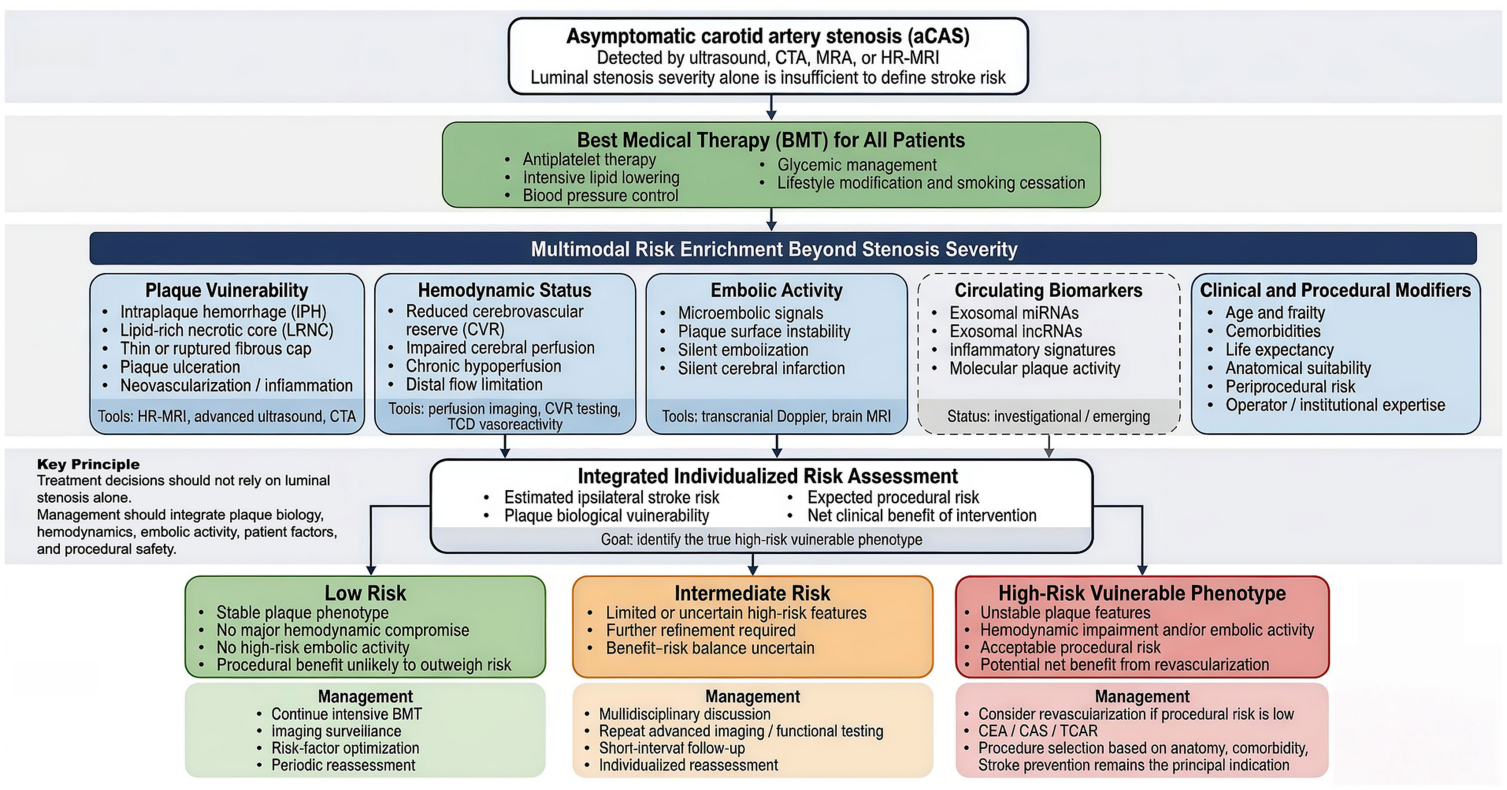
Individualized precision risk stratification framework for asymptomatic carotid artery stenosis. Clinical application of multimodal biomarkers should be guided by risk stratification. Standard carotid ultrasound and routine blood tests are indicated for all patients with aCAS. Advanced imaging (HR-MRI, perfusion imaging, CVR testing, TCD) and liquid biopsy (exosomal miRNAs/lncRNAs) should be reserved for selected subgroups, including those with: Ultrasound features suggestive of plaque vulnerability (hypoechoic plaque, ulceration, rapid progression); Prior contralateral stroke or TIA; Uncertain risk–benefit balance for revascularization; Younger age or life expectancy >5 years. PET imaging may be considered in research settings to assess plaque metabolic activity.

Collectively, these findings support a shift from stenosis-based decision-making toward a biologically informed precision framework. In this model, best medical therapy provides the foundation of care, while plaque vulnerability, cerebral hemodynamics, embolic activity, circulating biomarkers, and procedural factors are integrated to guide individualized management, as summarized in Figure 1.

#### Nature of this article and search strategy

This article is a critical narrative review, not a formal systematic review or meta-analysis. To identify relevant literature, we searched PubMed, Embase, and the Cochrane Library for English-language articles published between January 2021 and March 2026, using the following keywords: “asymptomatic carotid stenosis”, “best medical therapy”, “carotid endarterectomy”, “carotid artery stenting”, “transcarotid revascularization”, “high-resolution MRI”, “plaque vulnerability”, “exosomal microRNA”, and “cognitive impairment.” Reference lists of included articles and key trials (ACST-2, SPACE-2, ECST-2, CREST-2) were also screened. Although our primary focus is on contemporary evidence (2021–2026), we acknowledge that longer-term outcomes from foundational trials (e.g., ACAS, ACST-1, CREST-1) remain relevant and are discussed where they inform current guideline benchmarks (e.g., perioperative risk <3%). No formal risk-of-bias assessment was performed, which is a limitation of the narrative review format.

### Microstructural remodeling and paradigm evolution of best medical therapy

Current authoritative international guidelines in vascular surgery and neurology, including the 2023 European Society for Vascular Surgery (ESVS) guidelines and the 2022 Society for Vascular Surgery (SVS) guidelines, consistently assign BMT the highest level of recommendation (Class I, Level A/B) as the first-line foundational strategy for patients with aCAS (4, 5). To understand the evolution of BMT between 2021 and 2026, it is necessary to move beyond the traditional concept of isolated biochemical target control. More importantly, BMT should be examined from the perspectives of molecular biology and microstructural pathology. This helps clarify the pivotal role of contemporary pharmacological interventions in reshaping and stabilizing the biological phenotype of atherosclerotic plaques.

### The targeted era of lipid-lowering therapy: plaque-reversing effects of PCSK9 inhibitors

Low-density lipoprotein cholesterol (LDL-C) is a key initiating factor in atherogenesis. After oxidative modification, oxidized LDL (ox-LDL) is taken up by macrophages and promotes foam cell formation. These foam cells subsequently become a major component of the lipid-rich necrotic core (LRNC) within atherosclerotic plaques (6, 7). A randomized controlled trial showed that, in patients with atherosclerotic cardiovascular disease, sustained reduction of LDL-C to an extremely low level of <20 mg/dL (<0.5 mmol/L) not only reduced the risk of cardiovascular events but was also not associated with significant safety concerns (8). Although conventional high-intensity statin therapy can substantially lower LDL-C levels, considerable residual cardiovascular risk persists in some high-risk patients. In addition, long-term high-dose use is often accompanied by adverse effects such as myopathy and liver function abnormalities. In recent years, the widespread clinical use of proprotein convertase subtilisin/kexin type 9 (PCSK9) inhibitors has marked the formal entry of lipid-lowering therapy into the era of biologically targeted intervention (9).

PCSK9 inhibitors, such as evolocumab and alirocumab, specifically bind circulating PCSK9 through monoclonal antibody technology. This prevents its interaction with low-density lipoprotein receptors (LDLRs) on the hepatocyte surface and markedly inhibits the lysosomal degradation of LDLRs. As a result, the density of LDLRs on hepatocyte membranes increases, and the clearance of circulating LDL-C is substantially enhanced (10). Studies published between 2021 and 2026 have further shown that PCSK9 inhibitors may also promote plaque regression at the microscopic pathological level in aCAS. One observational study found that patients receiving PCSK9 inhibitors had lower expression of proinflammatory proteins and higher intraplaque levels of SIRT3 and collagen, despite comparable circulating hs-CRP levels. These findings remained evident in a matched subgroup with LDL-C < 100 mg/dL. After adjustment for multiple variables, including LDL-C, the risk of outcome events remained significantly lower in the PCSK9 inhibitor group than in patients receiving other lipid-lowering therapies (adjusted HR 0.262; 95% CI 0.131–0.524; *p* < 0.001). The study further showed that PCSK9 expression was positively correlated with proinflammatory proteins. It also showed that the inflammatory burden reflected by these proteins was associated with an increased risk of adverse outcomes, independent of treatment strategy. Taken together, these findings suggest that PCSK9 inhibitors can favorably remodel the inflammatory burden of atherosclerosis. This effect may be at least partly independent of LDL-C lowering and may provide additional cardiovascular benefit (11, 12).

The Phase 4 SLICE-CEA CardioLink-8 trial, presented at the 2024 European Society of Cardiology (ESC) Annual Congress, provided high-quality imaging evidence for the plaque-modifying effect of PCSK9 inhibitors. This study included patients with severe atherosclerotic coronary artery disease (ASCAD) and high-risk plaque features, with a stenosis rate of 70–99%. Evolocumab was added to standard treatment. Follow-up high-resolution magnetic resonance imaging (HR-MRI) showed that, 6 months after treatment, the volume of the lipid-rich necrotic core (LRNC) in the treatment group had decreased significantly. This change effectively slowed the progression of plaques toward a vulnerable phenotype (13). Furthermore, a longitudinal cohort study involving 131 patients with high-risk atherosclerosis, with a mean follow-up of 6 years, confirmed that within the first 3 years after initiation of PCSK9 inhibitor therapy, the total carotid plaque area (TPA) showed a continuous and statistically significant reduction.

It is worth noting that the clinical benefits of PCSK9 inhibitors are not limited to simple lipid clearance. Their anti-inflammatory pleiotropic effects also play a central role in stabilizing vulnerable plaques. Mechanistic studies have shown that alirocumab can significantly downregulate the serum expression levels of matrix metalloproteinase-9 (MMP-9), osteopontin (OPN), and osteoprotegerin (OPG), all of which are closely related to plaque rupture (14). MMP-9 is mainly secreted by inflammatory macrophages within the plaque. It promotes plaque instability by degrading collagen fibers in the fibrous cap matrix. Therefore, inhibition of MMP-9 activity helps preserve or increase fibrous cap thickness. This process may drive the transformation of structurally fragile plaques into more stable plaques at the biochemical level. This profound remodeling of plaque biology is a key molecular basis by which modern BMT can reduce the annual stroke risk in patients with aCAS to below 1%.

### Cardiovascular benefits of novel glucose-lowering agents beyond glycemic control: synergistic effects of SGLT-2 inhibitors and GLP-1 receptor agonists

Diabetes mellitus is a major driver of carotid atherosclerotic progression. Chronic hyperglycemia markedly worsens oxidative stress and chronic low-grade inflammation in the vascular endothelium by promoting the excessive accumulation of advanced glycation end products (AGEs) (15). Between 2021 and 2026, research at the interface of cardiovascular and metabolic medicine showed that sodium-glucose cotransporter 2 (SGLT-2) inhibitors, such as empagliflozin, and glucagon-like peptide-1 (GLP-1) receptor agonists, such as liraglutide and semaglutide, have effects that extend beyond glucose lowering. These agents provide significant cardiovascular protection that is at least partly independent of glycemic control (16).

A prospective interventional cohort study published in the American Journal of Physiology in 2025 systematically evaluated the clinical value of these agents in delaying carotid vascular aging and reducing plaque burden. The study enrolled 183 patients with type 2 diabetes mellitus. Using propensity score matching, it compared the effects of insulin, GLP-1 receptor agonist therapy, SGLT-2 inhibitor monotherapy, and their combined use on carotid intima-media thickness (cIMT), circulating amyloid beta 1–40 (Aβ1-40), and malondialdehyde (MDA), a biomarker of lipid peroxidation. After 12 months of follow-up, cIMT showed only a modest reduction of 1.7% in the insulin group. In contrast, reductions of 8.2 and 5.6% were observed in the liraglutide and empagliflozin groups, respectively. The combination therapy group achieved the greatest regression, with a 10.7% reduction in cIMT (*p* < 0.05). In addition, combination therapy showed the strongest effect in lowering Aβ1-40, a marker of endothelial injury, with a reduction of 50.7%, compared with 30.7% in the insulin group.

Mechanistically, GLP-1 receptor agonists appear to exert antiatherosclerotic effects mainly by inhibiting monocyte/macrophage adhesion and chemotaxis toward the vascular endothelium and by downregulating local proinflammatory mediators. In contrast, SGLT-2 inhibitors improve hemodynamic parameters through their osmotic diuretic effect, reduce abnormal mechanical shear stress on the vessel wall, and induce metabolic reprogramming toward ketone body utilization in the heart and kidney. The complementary and synergistic actions of these two drug classes at the levels of molecular signaling and hemodynamic regulation may contribute to a more stable plaque phenotype in patients with aCAS complicated by metabolic syndrome (17).

### Environmental exposures as emerging contributors to residual plaque risk: microplastics and nanoplastics

Although intensive pharmacological therapy has transformed the management of aCAS, optimized control of conventional risk factors does not eliminate all vascular risk. In some patients, plaque inflammation and vulnerability persist despite adequate LDL-C reduction, glycemic control, and blood-pressure management. This observation has encouraged growing interest in nontraditional contributors to residual plaque risk, including environmental exposures capable of sustaining vascular inflammation. Among these, microplastics and nanoplastics have recently attracted particular attention.

A prospective cohort study published in the New England Journal of Medicine in 2024 provided the first direct clinical evidence supporting the pathological relevance of microplastics and nanoplastics (MNPs) as an independent emerging cardiovascular risk factor (18). Marfella and colleagues (NCT05900947) conducted a multicenter observational study involving 304 patients with severe aCAS who underwent carotid endarterectomy (CEA). Using pyrolysis–gas chromatography–mass spectrometry (Py-GC/MS), stable isotope analysis, and high-resolution electron microscopy (EM), the investigators characterized the chemical composition and ultrastructural features of excised plaques. Among the 257 patients who completed follow-up, polyethylene was detected in 58.4% (150/257) of plaques at a concentration of 21.7 ± 24.5 μg/mg, whereas polyvinyl chloride (PVC) was identified in 12.1% (31/257) at a concentration of 5.2 ± 2.4 μg/mg. Ultrastructural imaging showed that jagged-edged polymer particles were mainly located within plaque macrophages, namely foam cells, as well as in the extracellular necrotic lipid matrix. After a mean follow-up of approximately 34 months, survival analysis further showed a strong association between intraplaque MNP burden and adverse prognosis. The incidence of major adverse cardiovascular events, defined as a composite of nonfatal myocardial infarction, nonfatal stroke, or death from any cause, was only 7.5% (8/107) in patients without detectable MNPs. In contrast, it increased to 20.0% (30/150) in those with detectable MNPs. After rigorous multivariable adjustment for age, sex, diabetes, hypertension, and prior cardiovascular disease, intraplaque MNP deposition remained associated with a 4.53-fold increase in the risk of major adverse cardiovascular events (hazard ratio, 4.53; 95% CI, 2.00–10.27; *p* < 0.001).

At the mechanistic level, enzyme-linked immunosorbent assay (ELISA) and immunohistochemical analyses showed that plaques enriched with MNPs had significantly higher expression of proinflammatory cytokines, including interleukin-6 (IL-6), interleukin-1β (IL-1β), interleukin-18 (IL-18), and tumor necrosis factor-*α*, than plaques without detectable MNPs. These nanoscale foreign particles may act as potent immunogenic stimuli. They may trigger persistent inflammatory activation and oxidative stress in local macrophages, thereby accelerating fibrous cap degradation and promoting plaque rupture. Although this observational study cannot establish definite causality and is subject to the selection bias inherent in surgically derived specimens, the magnitude of the reported association, reflected by a hazard ratio of 4.53, is striking in the context of cardiovascular epidemiology. These findings highlight microplastics and nanoplastics as emerging residual risk factors of interest, although causality remains unproven and the pathophysiological mechanisms are still under investigation. At present, there is no evidence-based strategy to clinically mitigate MNP exposure, and routine BMT does not include environmental risk modification. Nevertheless, this association underscores the need for further research into non-traditional drivers of plaque inflammation and for broader public health awareness regarding environmental exposures (19).

### Clinical decision-making dilemmas in revascularization: an evidence matrix from four pivotal RCTs published between 2021 and 2026

As best medical therapy (BMT) has reduced the natural risk of stroke in patients with aCAS to a relatively low level, the question of whether prophylactic revascularization justifies its accompanying periprocedural risk has become increasingly important between 2021 and 2026. Four pivotal randomized controlled trials (RCTs), namely ACST-2, SPACE-2, ECST-2, and CREST-2, have together formed the contemporary high-level evidence base for clinical decision-making. These studies have supported the role of revascularization in selected settings. At the same time, they have also raised important challenges to the traditional framework used to define procedural indications (Table 1).

**Table 1:** Major randomized controlled trials informing contemporary management of asymptomatic carotid artery stenosis, 2021–2026.

Trial, year	Study population	Sample size	Treatment comparison	Primary endpoint/main outcome	Key findings	Interpretation
ACST-2, 2021	Severe aCAS suitable for CAS or CEA	3,625	CAS vs. CEA	Procedural death or disabling stroke; long-term nonprocedural stroke	CAS and CEA showed similar long-term outcomes. Procedural death or disabling stroke at 30 days was 1.0% with CAS and 0.9% with CEA; 5-year procedural death or disabling stroke was 3.4 and 3.5%, respectively.	ACST-2 supports the procedural equivalence of CAS and CEA in selected patients, but it does not determine whether revascularization is superior to contemporary medical therapy because there was no BMT-only arm.
SPACE-2, 2022	Moderate-to-severe aCAS	513	CEA + BMT vs. CAS + BMT vs. BMT alone	Any stroke or death within 30 days, or ipsilateral ischemic stroke within 5 years	The 5-year primary endpoint occurred in 2.5% with CEA + BMT, 4.4% with CAS + BMT, and 3.1% with BMT alone. No clear superiority of revascularization was demonstrated.	SPACE-2 suggests that contemporary BMT may provide low stroke risk, but the trial was underpowered because of premature termination and limited recruitment.
ECST-2, 2025	Low- to intermediate-risk carotid stenosis	429	OMT alone vs. OMT + revascularization	Hierarchical composite outcome analyzed by win ratio	The 2-year interim analysis showed no evidence of benefit from adding revascularization to OMT; win ratio 1.01, 95% CI 0.60–1.70, *p* = 0.97.	ECST-2 supports a medicine-first strategy in low-to-intermediate-risk patients, while longer follow-up is needed before applying the findings to all severe aCAS patients.
CREST-2, 2025/2026	Severe aCAS ≥70%	2,485	CAS + IMM vs. IMM; CEA + IMM vs. IMM	Any stroke or death within 44 days, or ipsilateral ischemic stroke within 4 years	In the stenting trial, the primary outcome occurred in 2.8% with CAS + IMM vs. 6.0% with IMM alone, *p* = 0.02. In the endarterectomy trial, it occurred in 3.7% with CEA + IMM vs. 5.3% with IMM alone, *p* = 0.24.	CREST-2 suggests that CAS may provide additional benefit under highly controlled procedural conditions, whereas CEA did not reach statistical superiority. The findings support selective, rather than routine, revascularization.

When considering the choice of revascularization strategy for aCAS, namely carotid artery stenting (CAS) versus carotid endarterectomy (CEA), the Second Asymptomatic Carotid Surgery Trial (ACST-2), published in The Lancet in 2021, provided key high-level evidence (20). As the largest international multicenter RCT to date comparing CAS with CEA in patients with aCAS, ACST-2 enrolled 3,625 patients with severe asymptomatic carotid stenosis (≥60%) from 130 centers across 33 countries between 2008 and 2020. Patients were randomly assigned in a 1:1 ratio to the CAS group (n = 1811) or the CEA group (n = 1814). They were then followed for a mean of 5 years under standardized contemporary medical therapy. The trial showed a high degree of safety and clinical equivalence between the two procedures. First, with respect to periprocedural safety within 30 days, the rate of disabling stroke or death was approximately 1% in both groups (15 cases in the CAS group and 18 in the CEA group), with no statistically significant difference. The overall incidence of nondisabling periprocedural stroke was also around 2% (48 cases in the CAS group vs. 29 in the CEA group). These findings indicate that, under contemporary perioperative management and in the hands of experienced operators, both CAS and CEA can achieve procedural safety that meets, and remains below, the 3% threshold recommended by international guidelines. Second, regarding long-term protective efficacy over 5 years, Kaplan–Meier survival analysis showed that the cumulative probability curves for any nonprocedural stroke were very similar between the two groups. The estimated 5-year incidence was 5.3% in the CAS group and 4.5% in the CEA group (rate ratio, 1.16; 95% CI, 0.86–1.57; *p* = 0.33), with no significant between-group difference. For the key endpoint of fatal or disabling stroke, the estimated 5-year incidence was identical in the two groups at 2.5%.

In addition, the 10-year extension data from ACST-2, presented at the 2025 annual meeting of the European Society for Vascular Surgery (ESVS), further supported these conclusions. The incidence of first stroke remained highly similar between the two groups (113 cases in the CAS group vs. 112 in the CEA group). Moreover, most stroke events arose from untreated contralateral vascular lesions. In contrast, the incidence of ipsilateral stroke in the target vessel was substantially lower than that of contralateral stroke (46 vs. 73), supporting the durable protective effect of both procedures on the treated artery (21). The major academic contribution of ACST-2 lies in its systematic response to earlier concerns about the long-term efficacy of CAS. It established that, in appropriately selected patients with favorable anatomy, CAS can serve as a safe, minimally invasive, and clinically equivalent alternative to CEA (22, 23).

### Reappraising prophylactic intervention: evidence-based insights from SPACE-2 and ECST-2

Although ACST-2 clarified the relative performance of the two revascularization techniques, its study design had one important limitation: the absence of a best medical therapy (BMT)-only control group (24). As a result, it did not directly answer the central clinical question of whether prophylactic revascularization remains necessary in the era of contemporary intensive medical therapy. To address this gap, European investigators subsequently initiated two pivotal randomized controlled trials, SPACE-2 and ECST-2.

The SPACE-2 trial (Stent-Protected Angioplasty versus Carotid Endarterectomy-2) was originally designed as a three-arm RCT comparing carotid endarterectomy (CEA), carotid artery stenting (CAS), and BMT alone. However, because of practical challenges such as strict enrollment criteria and increasing clinical preference for modern BMT, patient recruitment was difficult. The trial was therefore terminated early in 2022 after enrolling 513 patients, far below the planned sample size. Although early termination inevitably reduced its statistical power, the 5-year follow-up data published in The Lancet Neurology remain highly informative. No statistically significant differences were observed among the CAS, CEA, and BMT-alone groups for the primary composite endpoint, which included periprocedural stroke or death and long-term ipsilateral ischemic stroke. No significant difference was also seen in the overall stroke rate (25). Although SPACE-2 did not provide a definitive answer, its preliminary findings clearly suggest that the stroke-preventive effect of contemporary BMT may have been underestimated in earlier studies.

Following SPACE-2, the Second European Carotid Surgery Trial (ECST-2), published in The Lancet Neurology in 2025, posed an even greater challenge to the necessity of prophylactic intervention (26). ECST-2 was a large multicenter RCT conducted across Europe and Canada. It primarily enrolled patients with low- to intermediate-risk aCAS, as well as patients with recent minor symptoms, whose predicted 5-year risk of ipsilateral stroke was below 20% according to the Carotid Artery Risk (CAR) score. Participants were randomly assigned to receive either optimized medical therapy (OMT) alone or OMT combined with revascularization, including either CEA or CAS. Methodologically, the study was notable for its use of the win ratio, a statistical method designed to analyze complex hierarchical composite endpoints. The 2-year interim analysis showed no advantage for either strategy, with a win ratio of 1.01 (95% CI, 0.60–1.70; *p* = 0.97). The win ratio is a statistical method that hierarchically compares pairs of patients for a composite endpoint (e.g., death, stroke, myocardial infarction, then silent infarction), giving priority to more severe events. For clinical interpretation, the absolute risk of the primary composite endpoint was also similar between groups: approximately 6% in the OMT-alone group and 5.9% in the revascularization group, corresponding to an absolute risk difference of −0.1% (95% CI, −2.9 to 2.7%). These findings indicate a high degree of clinical equivalence between OMT alone and OMT plus revascularization for composite outcomes including periprocedural cardiovascular and cerebrovascular events and newly detected silent cerebral infarction (27). Further analysis showed that adding revascularization did not further reduce stroke risk. Instead, it introduced procedure-related periprocedural complications, including one death secondary to postoperative decompensation of aortic stenosis. The interim results of ECST-2 deliver a clear clinical message: in patients with aCAS who are classified as low to intermediate risk by validated risk models, adding revascularization does not appear to provide a net clinical benefit over 2 years of short-term follow-up. At present, optimized medical therapy alone should therefore be regarded as the preferred strategy for this population.

### Breaking the deadlock and redefining the paradigm: an in-depth analysis of CREST-2

The pivotal randomized controlled trial designed to determine whether modern best medical therapy (BMT) or prophylactic revascularization provides greater benefit, the Carotid Revascularization and Medical Management for Asymptomatic Carotid Stenosis Trial (CREST-2), was recently formally published in the New England Journal of Medicine by James F. Meschia, Thomas G. Brott, and colleagues. This study provides what is arguably the most decisive high-level evidence to date for this long-standing controversy (28). Methodologically, CREST-2 used an innovative design that avoided the loss of statistical efficiency often seen in traditional three-arm trials. Instead, it consisted of two independent, parallel randomized trial cohorts. The stenting trial compared carotid artery stenting (CAS) plus intensive medical management (IMM) with IMM alone (*n* = 1,245). The endarterectomy trial compared carotid endarterectomy (CEA) plus IMM with IMM alone (*n* = 1,240). Eligible participants had severe carotid stenosis (≥70%) and had remained asymptomatic during the preceding 6 months. The primary composite endpoint was defined as any stroke or death from randomization through day 44, together with ipsilateral ischemic stroke during the subsequent follow-up period of up to 4 years.

The results of CREST-2 have important implications for current management strategies in aCAS. In the stenting trial, after 4 years of follow-up, the incidence of the primary endpoint was 6.0% (95% CI, 3.8–8.3) in the IMM-alone group, compared with 2.8% (95% CI, 1.5–4.3) in the CAS plus IMM group. This absolute risk difference was statistically significant (*p* = 0.02). This finding indicates that, even in the setting of contemporary intensive medical therapy, high-quality CAS can provide additional stroke-preventive benefit in patients with severe aCAS. In contrast, in the endarterectomy trial, the primary endpoint occurred in 5.3% (95% CI, 3.3–7.4) of patients assigned to IMM alone and in 3.7% (95% CI, 2.1–5.5) of those assigned to CEA plus IMM. Although the numerical trend favored surgery, the between-group difference did not reach statistical significance (*p* = 0.24). The accompanying interpretation in NEJM was therefore clear: carotid endarterectomy did not result in a significant benefit under the conditions tested in this trial.

From a deeper pathophysiological and methodological perspective, the finding that CAS achieved superiority whereas CEA did not reach the primary endpoint may appear, at first glance, to challenge earlier paradigms. These include the equivalence observed in ACST-2 and the relative advantage previously suggested for CEA in some earlier settings. In reality, these results are more plausibly explained by both technical evolution and trial design. First, operator qualification in CREST-2 was exceptionally stringent. Among the 334 interventionists who initially applied, only 9% were approved at initial review. The final approval rate reached 46% only after additional training, submission of further cases, and repeat review. This rigorous credentialing framework, together with contemporary embolic protection strategies and improved stent technology, likely contributed to the very low 0-to-44-day stroke-or-death rate of 1.3% in the stenting arm.

Second, the results of the endarterectomy trial can be interpreted in the context of a front-loaded offset effect. Although CEA may still reduce later ipsilateral stroke risk, the invasiveness of open surgery is associated with a higher burden of early procedural events. In CREST-2, during the first 44 days, 9 strokes occurred in the CEA group, compared with 3 in the IMM-alone group. Because the primary analysis assigned equal statistical weight to early procedural stroke and later stroke during natural follow-up, these early events substantially diluted the long-term preventive signal of surgery. In addition, subsequent methodological commentaries have noted that perioperative myocardial infarction was not included in the primary composite endpoint of CREST-2, in contrast to CREST-1. Because myocardial infarction has historically occurred more often after CEA than after CAS, inclusion of this endpoint might have further narrowed the apparent net clinical benefit of CEA.

Taken together, CREST-2 does not completely negate the clinical value of CEA. Rather, it suggests that carotid revascularization in aCAS has entered an era in which net benefit depends on extremely low procedural risk, precise anatomic selection, and highly experienced operators. Based on currently available randomized evidence, prophylactic CAS is the only revascularization strategy that has demonstrated superiority over contemporary intensive medical therapy in patients with severe aCAS under rigorously controlled trial conditions.

### Transcarotid artery revascularization: a real-world evidence-based strategy for patients with high-risk anatomy

While the efficacy of conventional carotid artery stenting (CAS) and carotid endarterectomy (CEA) continued to be evaluated, clinical practice between 2021 and 2026 also saw the growing adoption of transcarotid artery revascularization (TCAR), an innovative hybrid approach to carotid intervention. In elderly patients with marked aortic arch tortuosity, including bovine arch configuration or type III arch, or in those with heavily calcified plaques, conventional transfemoral CAS (tfCAS) may require extensive catheter and guidewire manipulation. This may increase the risk of plaque disruption and periprocedural embolic stroke. TCAR was developed in part to avoid these access-related anatomic hazards. Through a small supraclavicular incision, the common carotid artery is directly exposed to create a short access route. Its key technical feature is a dynamic flow-reversal system. In this system, cerebral embolic protection is achieved by reversing carotid flow during the critical phases of stent deployment and directing blood through an extracorporeal circuit to the femoral venous system, thereby limiting distal embolization into the intracranial circulation (29). Although large randomized controlled trials directly comparing TCAR with best medical therapy (BMT) alone are still lacking, substantial real-world evidence from the Society for Vascular Surgery Vascular Quality Initiative (VQI), VISION, and related datasets has helped fill part of this evidentiary gap.

A 2024 systematic review and study-level meta-analysis published in Stroke included 24,246 patients who underwent carotid revascularization, including 4,771 treated with TCAR, 12,350 with CEA, and 7,125 with CAS (30). Additional real-world evidence has further strengthened the case for TCAR in anatomically complex patients. A 2025 comparative effectiveness analysis published in JAMA Network Open showed that, among both asymptomatic and symptomatic patients undergoing carotid stenting, TCAR was associated with a significantly lower long-term risk of stroke than tfCAS. This benefit remained durable over 3 years of follow-up. Among asymptomatic patients, the 3-year risk of stroke was 5.1% after TCAR and 9.2% after tfCAS, with an adjusted hazard ratio of 1.69 for tfCAS relative to TCAR (31). These findings are particularly relevant for older patients and for those with unfavorable arch anatomy, in whom transfemoral access itself may contribute substantially to procedural risk.

On the basis of these safety and effectiveness data, the 2022 Society for Vascular Surgery (SVS) guidelines state that patients with asymptomatic carotid stenosis of at least 70% and a life expectancy of 3 to 5 years may be considered for carotid intervention, including CEA, TCAR, or transfemoral CAS, provided that the expected perioperative risk of stroke and death does not exceed 3%. The choice of procedure should be individualized according to the presence or absence of high-risk features for each specific intervention. In clinical practice, factors that increase the technical difficulty of CEA, such as a high carotid bifurcation, prior neck irradiation, or contralateral vocal cord paralysis, are often among the anatomic considerations that favor less invasive alternatives in appropriately selected patients. Nevertheless, it should be acknowledged that current support for TCAR in aCAS still relies predominantly on high-quality observational evidence rather than direct head-to-head randomized comparisons with BMT alone (32, 33).

### Paradigm evolution in risk stratification: toward a precision assessment framework beyond anatomic stenosis

Taken together, the findings of ECST-2 and CREST-2 have led to a growing consensus that the degree of luminal stenosis measured by two-dimensional ultrasonography or angiography alone, such as 70% or 80%, is no longer sufficient as an independent basis for revascularization decisions. Many patients with severe stenosis may already have heavily calcified and biologically stable plaques. In such patients, unnecessary intervention may instead expose them to avoidable periprocedural risk. Accordingly, the clinical management of aCAS is moving toward a precision risk stratification framework based on multimodal imaging and molecular biomarkers. The aim is to characterize the underlying plaque biology noninvasively and to identify the small subset of truly high-risk vulnerable patients within the much larger population of clinically stable individuals.

Among the currently available noninvasive imaging modalities, high-resolution magnetic resonance imaging (HR-MRI), particularly when combined with 3D black-blood and contrast-enhanced sequences, has gradually emerged as a key tool for assessing carotid plaque vulnerability (34). HR-MRI allows high-resolution qualitative and quantitative assessment of intraplaque tissue composition. Several imaging features have shown substantial value in stroke risk prediction. Intraplaque hemorrhage (IPH) arises pathologically from proliferation of the vasa vasorum and rupture of fragile neovessels within the plaque. Because these newly formed microvessels are structurally abnormal and have loose endothelial junctions, erythrocytes may leak into the lipid-rich necrotic core and degrade into methemoglobin. On T1-weighted MRI sequences such as MP-RAGE, IPH typically appears as a hyperintense signal. Large meta-analyses based on individual patient data published between 2021 and 2024 have shown that IPH is a strong independent predictor of future ischemic stroke (35). In patients with aCAS, the risk of ipsilateral stroke in the presence of MRI-detected IPH is 5.9- to 11.0-fold higher than in those without IPH (HR = 11.0, 95% CI: 4.8–25.1). Importantly, this predictive value is independent of conventional vascular risk factors and the severity of luminal narrowing. Compared with a stable and heavily calcified plaque causing 90% stenosis, a plaque causing only 60% stenosis but accompanied by active IPH may carry a substantially higher risk of acute thrombotic events.

A large lipid-rich necrotic core (LRNC) is also closely associated with plaque biomechanical instability and local inflammatory burden. Previous pathological and imaging studies have suggested that when the LRNC occupies more than 40% of total plaque volume, the plaque structure is often close to a critical threshold of instability. A thin or ruptured fibrous cap (TRFC) is another major imaging marker of plaque vulnerability. The collagen-rich fibrous cap overlying the LRNC is the main physical barrier that prevents thrombogenic material from being exposed to the bloodstream. On contrast-enhanced HR-MRI, interruption or loss of the continuous enhancing surface signal suggests TRFC. Meta-analytic data have shown that the presence of TRFC is associated with a marked increase in the risk of ipsilateral stroke or transient ischemic attack (TIA) (HR = 5.93).

To standardize the assessment of carotid plaque vulnerability, the Carotid Plaque-RADS score has recently been introduced as a comprehensive imaging algorithm. It categorizes plaques based on intraplaque hemorrhage, lipid-rich necrotic core, fibrous cap status, and calcification patterns, providing a structured framework for clinical reporting and risk stratification. This scoring system may facilitate more consistent decision-making across centers and trials.

In view of the clear value of HR-MRI in risk stratification, recent international clinical consensus documents, including the 2023 ESVS guidelines, have recommended that high-risk imaging features such as IPH and a large LRNC should be taken into account when evaluating the need for intervention in patients with 60–99% aCAS (36). At present, AI-assisted radiomics models based on HR-MRI features, such as the IMPROVE risk scoring system, are under active development and validation. In the future, they may provide more precise and individualized stroke risk prediction curves for patients with aCAS (37).

### Liquid biopsy: exosomes and noncoding RNAs at the frontier of molecular diagnosis

In parallel with advances in imaging technologies, liquid biopsy has made substantial progress between 2021 and 2026 as a simple, early, and completely noninvasive approach to risk stratification. Research in this field has focused mainly on extracellular vesicles, particularly exosomes measuring 40–160 nm in diameter, and on the post-transcriptional regulatory molecules they carry, especially microRNAs (miRNAs) and long noncoding RNAs (lncRNAs) (38).

Exosomes are no longer viewed simply as vesicles for the disposal of cellular metabolic byproducts. They are now recognized as important mediators of intercellular communication among vascular endothelial cells, macrophages, and smooth muscle cells through both endocrine and paracrine pathways. Because their lipid bilayer membrane provides tight encapsulation, exosome-derived RNAs show strong resistance to nuclease degradation in the circulation. This makes them naturally stable and highly promising circulating biomarkers (39).

Peripheral blood transcriptomic studies in patients with aCAS have revealed a complex network of inflammatory regulation at the microscopic level. In patients with aCAS accompanied by vascular remodeling and plaque progression, several exosomal miRNAs, including miR-199b-3p, miR-27b-3p, miR-130a-3p, miR-221-3p, and miR-24-3p, have been found to be significantly upregulated (*p* < 0.05). Functional validation studies further suggest that these miRNAs are closely involved in the suppression of endothelial nitric oxide synthase (eNOS) activity and the abnormal activation of the NLRP3 inflammasome. In addition, in cohorts of patients with aCAS and concomitant type 2 diabetes mellitus, a combined logistic regression model based on four serum exosomal miRNAs, hsa-miR-433-3p, hsa-let-7b, hsa-miR-30-5p, and hsa-miR-122-5p, showed excellent predictive performance for the diagnosis of subclinical severe carotid atherosclerosis (40).

In cohorts of patients with large artery atherosclerotic (LAA) stroke, deep RNA sequencing (RNA-seq) has identified several prognostically relevant exosomal lncRNAs, including exo-lnc_000048, exo-lnc_001350, and exo-lnc_016442. In univariable analyses, each of these biomarkers achieved an area under the curve (AUC) greater than 0.82. When these three lncRNAs were combined with the National Institutes of Health Stroke Scale (NIHSS) score in a composite prediction model, the AUC for predicting stroke severity and long-term unfavorable functional outcomes increased to 0.936. This performance was markedly better than that of conventional biochemical indicators such as total cholesterol (TC) and triglycerides (TG) (41).

Looking ahead, exosomal molecular fingerprinting based on high-throughput sequencing may become part of future screening pathways for high-risk aCAS populations. By analyzing small-volume peripheral blood samples with the support of artificial intelligence (AI) and machine learning algorithms, clinicians may be able to quantify the inflammatory burden and autophagic status of target-vessel plaques at the molecular level (42). The establishment of such a diagnostic framework could allow precise stroke warning before macroscopic anatomic abnormalities become visible on imaging. This would shift the clinical management of aCAS from passive morphologic surveillance toward truly early and individualized molecular intervention.

It is important to acknowledge that most advanced imaging and molecular biomarkers are not yet widely available or reimbursed in routine clinical practice. Therefore, their use should follow a stepwise, resource-conscious approach, reserving high-cost modalities for patients in whom the decision for or against revascularization remains uncertain after standard assessment.

#### Reconstructing the clinical definition of “asymptomatic”: cognitive decline and evidence from the CREST-H study

In traditional neurological assessment, symptom status has often been defined in an “all-or-none” binary manner. Patients are generally classified as asymptomatic if they do not show focal motor deficits such as hemiparesis, aphasia, or transient monocular blindness. However, the brain is a highly energy-dependent organ and is particularly vulnerable to hypoxia. For this reason, the possible subclinical effects of chronic hypoperfusion and recurrent microembolization caused by severe carotid stenosis have received increasing attention.

In recent years, several systematic reviews and large-scale cohort studies have substantially challenged the traditional definition of “asymptomatic.” Between 2021 and 2026, epidemiological evidence from real-world claims data involving more than 100,000 individuals, together with longitudinal follow-up, showed that even after strict exclusion of patients with a prior history of clinically overt stroke, those with severe aCAS had a 1.22-fold higher relative risk of Alzheimer’s disease (AD) and a 1.48-fold higher risk of non-Alzheimer dementia than age-matched healthy controls (43). The pathophysiological mechanisms underlying this covert brain injury and progressive cognitive decline are likely multifactorial and may act synergistically. Chronic hemodynamic impairment appears to play a central role. When carotid stenosis reaches ≥70%, distal intracranial perfusion pressure on the ipsilateral side declines substantially. Once the vasodilatory compensatory capacity of cerebrovascular reserve (CVR) is exhausted, the brain parenchyma remains in a state of sustained hypoperfusion. This can impair neuronal mitochondrial energy metabolism and trigger oxidative stress cascades, predominantly affecting the hippocampus and the frontoparietal cortical networks responsible for learning, memory, and information-processing speed. Silent microembolism may also make an important contribution. Small thrombotic fragments or atherosclerotic debris continuously shed from the surface of vulnerable plaques can enter the intracranial circulation. Although these emboli are too small to occlude major arteries and therefore do not produce classical focal neurological signs, they may cause multiple microinfarcts in the deep subcortical white matter. On MRI, this type of microvascular injury often appears as diffuse white matter hyperintensities (WMH). These lesions can disrupt neural connections between the cortex and subcortical nuclei and thereby impair executive function and attention (44, 45).

Taken together, these findings have prompted an important conceptual shift in contemporary cerebrovascular medicine. To a considerable extent, the traditional label of “asymptomatic” may reflect a diagnostic blind spot caused by the lack of sufficiently sensitive neuropsychological assessment tools. In patients with severe aCAS who already show progressive memory decline or executive dysfunction, the clinical phenotype may be more appropriately regarded as a high-risk stage equivalent to symptomatic disease.

### The controversy over reversing cognitive decline through intervention

Given that chronic ischemia is considered one of the initiating drivers of cognitive decline, an important question is whether relieving anatomic large-vessel stenosis through CEA or CAS and restoring cerebral perfusion can improve neuronal function and thereby reverse cognitive dysfunction (46).

Some earlier observational studies with relatively small sample sizes and short follow-up periods supported this hypothesis. For example, a cohort study published in Frontiers in Neurology in 2025 suggested that 3 to 12 months after CEA or CAS, patients showed statistically significant improvements in total Montreal Cognitive Assessment (MoCA) scores compared with preoperative baseline values, particularly in the domains of delayed recall and attention (*p* < 0.05). However, because such single-center observational studies lacked a parallel control group receiving medical therapy alone, the reliability of their conclusions remained methodologically limited (47). This hypothesis was subsequently not confirmed in a more rigorous randomized controlled trial setting.

As a nested cognitive substudy of the broader CREST-2 trial, CREST-H (Hemodynamics) has recently reported its key follow-up data. The study performed structured longitudinal neuropsychological assessments at baseline and during up to 4 years of follow-up, covering major cognitive domains including learning, attention, memory, and executive function. Baseline findings did confirm that patients with severe aCAS had significantly impaired cognitive performance compared with healthy controls, with lower Z scores for word-list recall in the memory domain (48). However, in the critical comparison of treatment effects, neither patients randomized to CAS nor those randomized to CEA showed any statistically significant improvement in longitudinal cognitive trajectories compared with the control group receiving intensive medical therapy alone (49).

These results indicate that prophylactic revascularization neither accelerates cognitive recovery nor slows cognitive decline. This negative finding provides important insight into the deeper neuropathological basis of vascular cognitive impairment. In patients with aCAS of ≥70% who already show cognitive deterioration, prolonged hemodynamic compromise may already have led to irreversible neuronal loss and structural disruption of neural networks (50, 51). Although mechanical revascularization can restore antegrade flow in large arteries, it cannot repair neurons that have already undergone irreversible degenerative injury. In addition, complex pathological cascades associated with aging, cerebral small vessel disease (CSVD), and chronic neuroinflammation cannot be fundamentally halted simply by relieving large-artery stenosis. The findings of CREST-H therefore define an important boundary for current clinical practice. Based on the existing high-level evidence, expanding the surgical indications for aCAS with the aim of improving or reversing cognitive decline is not scientifically justified. Prevention of ipsilateral focal ischemic stroke remains the only reasonable clinical indication for prophylactic revascularization. In the setting of potential vascular cognitive impairment, clinical management should shift earlier toward preventing plaque progression and microembolic shedding through standardized high-intensity medical therapy and lifestyle intervention before irreversible central nervous system injury occurs.

## Conclusions and future perspectives: toward an individualized precision medicine paradigm

Viewed against developments between 2021 and 2026, the management of asymptomatic carotid artery stenosis (aCAS) has gradually moved beyond the purely anatomy-based framework established in the late twentieth century. It is now shifting from macroscopic morphological correction toward intervention guided by underlying biological characteristics. With the publication of the pivotal CREST-2 and ECST-2 trials, high-level evidence has substantially challenged the traditional assumption that severe stenosis necessarily indicates high stroke risk and therefore warrants revascularization. In contemporary cardiovascular and metabolic medicine, intensified lipid lowering with PCSK9 inhibitors, metabolic regulation through the combined use of SGLT-2 inhibitors and GLP-1 receptor agonists, and attention to emerging environmental risk factors such as microplastics—although not yet clinically actionable—has broadened the scope of residual risk research and may inform future public health strategies. However, contemporary BMT remains centered on intensive lipid-lowering, metabolic control, antiplatelet therapy, and lifestyle intervention. These strategies have shown meaningful effects on plaque stabilization and phenotypic modification. For most patients with aCAS who are classified as low to intermediate risk after refined risk stratification, likely more than 80% of cases, standardized BMT alone may provide long-term outcomes comparable to or better than those of prophylactic intervention, while avoiding iatrogenic cardiovascular and cerebrovascular complications related to procedural trauma and microembolization.

This does not mean that prophylactic revascularization no longer has a role in the management of aCAS. Its indications, however, are becoming more selective and individualized. By showing the superiority of CAS under conditions of rigorous operator credentialing, CREST-2 delivered an important message: the long-term net clinical benefit of revascularization depends heavily on ultra-low procedural risk, meticulous technique, and effective perioperative neuroprotection. At the same time, the wider use of newer hybrid approaches such as transcarotid artery revascularization (TCAR) has provided an effective alternative for high-risk populations. This is particularly relevant for patients with complex vascular anatomy, such as marked aortic arch tortuosity, and for very elderly patients who may be poor candidates for conventional open surgery. In the setting of an aging population and a growing burden of vascular cognitive decline, the negative findings of CREST-H further suggest that delayed mechanical restoration of blood flow cannot reverse neuronal injury once it has become irreversible within complex central nervous system networks. Clinical management must therefore shift toward earlier intervention in the disease process.

When a patient is incidentally found to have severe aCAS (≥70%), the key clinical question is no longer simply whether revascularization should be performed. The priority is how to establish a precision risk assessment framework based on multimodal data. HR-MRI can be used to characterize plaque morphology at high resolution and identify high-risk features such as intraplaque hemorrhage (IPH) or a large lipid-rich necrotic core. Small-volume peripheral venous blood samples may also help quantify inflammatory burden and plaque destabilization through molecular biomarkers such as exosomal miRNAs and lncRNAs. Revascularization with CEA, CAS, or TCAR should be considered only when this multidimensional risk model identifies a truly high-risk vulnerable phenotype, or when standardized BMT fails to prevent plaque progression and ongoing microembolic activity. Even in such cases, the choice of revascularization strategy should remain highly individualized. CEA may be relatively advantageous in patients with heavily calcified plaques or marked vascular tortuosity that is unfavorable for endovascular access. CAS or TCAR may be more suitable for patients of advanced age, those with severe cardiopulmonary comorbidity, or those with a history of cervical irradiation.

The evolution of medicine is continuous. The shift from purely anatomic intervention to precise, biologically informed management reflects a deeper understanding of the true pathobiology of aCAS. In this process, close multidisciplinary collaboration among basic scientists, imaging specialists, and clinicians will be essential for building a future precision medicine framework centered on meaningful patient benefit.

### Limitations of this review

Several limitations should be acknowledged. First, as a narrative review, we did not perform a formal risk-of-bias assessment (e.g., Cochrane RoB 2) for the included trials, nor did we follow PRISMA guidelines for systematic reviews. Second, our deliberate focus on 2021–2026 may underrepresent long-term follow-up data from earlier trials, although key historical benchmarks are discussed. Third, many advanced imaging and molecular biomarkers discussed are not yet widely available or reimbursed; our proposed framework is therefore a conceptual model that requires prospective validation and health-economic evaluation. Fourth, the win ratio method used in ECST-2, while statistically robust, may be less intuitive for some clinicians; we have provided absolute risk differences where possible to aid interpretation. As highlighted by Abbott’s 2022 systematic review, the absence of reproducible, bias-limited methodologies can overestimate procedural benefits, underscoring the need for formal quality assessment in future evidence syntheses (52).
